# Explainable ML models for a deeper insight on treatment decision for localized prostate cancer

**DOI:** 10.1038/s41598-023-38162-1

**Published:** 2023-07-17

**Authors:** Jang Hee Han, Sungyup Lee, Byounghwa Lee, Ock-kee Baek, Samuel L. Washington, Annika Herlemann, Peter E. Lonergan, Peter R. Carroll, Chang Wook Jeong, Matthew R. Cooperberg

**Affiliations:** 1 Department of Urology, Seoul National University Hospital, Seoul, Republic of Korea; 2 Electronics and Telecommunications Research Institute (ETRI), Daejeon, Republic of Korea; 3 Department of Urology, Helen Diller Family Comprehensive Cancer Center, University of California, San Francisco, CA USA; 4 Department of Epidemiology and Biostatistics, University of California, San Francisco, CA USA; 5 Department of Urology, Ludwig-Maximilians-University of Munich, Munich, Germany; 6 Department of Urology, St. James’s Hospital, Dublin, Ireland; 7 Department of Surgery, Trinity College, Dublin, Ireland; 8 Department of Urology, Seoul National University College of Medicine, Seoul, Republic of Korea

**Keywords:** Health care, Medical research, Oncology, Urology

## Abstract

Although there are several decision aids for the treatment of localized prostate cancer (PCa), there are limitations in the consistency and certainty of the information provided. We aimed to better understand the treatment decision process and develop a decision-predicting model considering oncologic, demographic, socioeconomic, and geographic factors. Men newly diagnosed with localized PCa between 2010 and 2015 from the Surveillance, Epidemiology, and End Results Prostate with Watchful Waiting database were included (n = 255,837). We designed two prediction models: (1) Active surveillance/watchful waiting (AS/WW), radical prostatectomy (RP), and radiation therapy (RT) decision prediction in the entire cohort. (2) Prediction of AS/WW decisions in the low-risk cohort. The discrimination of the model was evaluated using the multiclass area under the curve (AUC). A plausible Shapley additive explanations value was used to explain the model’s prediction results. Oncological variables affected the RP decisions most, whereas RT was highly affected by geographic factors. The dependence plot depicted the feature interactions in reaching a treatment decision. The decision predicting model achieved an overall multiclass AUC of 0.77, whereas 0.74 was confirmed for the low-risk model. Using a large population-based real-world database, we unraveled the complex decision-making process and visualized nonlinear feature interactions in localized PCa.

## Introduction

Accounting for more than 75% of newly diagnosed men with prostate cancer (PCa)^1^, localized PCa exhibits remarkable inter-tumor heterogeneity and risk group diversity^2^; thus, various treatment options are now being proposed without solid decision criteria.

In fact, none of the possible treatments—such as observation (active surveillance(AS)/watchful waiting(WW)), radical prostatectomy (RP), or radiation therapy (RT)—have been proven superior in terms of cancer control in localized disease^3^, Thus, an initial treatment decision is often based on the patient’s PCa risk stratification and patient’s and physician’s treatment preference^4,5^. However, in the real-world setting, decision-making is a complex process that is not only affected by cancer characteristics but also by various patient-level, state/county-level regional, and socioeconomic factors^6^. Thus, a comprehensive approach is strongly needed for patients and physicians. In order to address this issue, there are several decision aids (DAs) for localized PCa patients^7^. However, there is lack of uniformity between these decision aids and their performances^8^.

An explainable machine learning model offers advantages by obtaining a deeper understanding of the internal processes, while the model itself trains or makes decisions and identifies cause-and-effect relationships within the system’s inputs and outputs^9^. In this study, we highlight the complex treatment decision process in localized PCa using the Surveillance, Epidemiology, and End Results (SEER) prostate with watchful waiting dataset (SEER/WW) through an explainable machine learning model. Using two-variable interaction plots, our primary aim was to gain a deeper insight into the important features associated with each treatment modality. Our secondary aim was to develop a treatment decision prediction model considering overall features, including oncologic, geographic (county-level data), demographic, and socioeconomic factors, which were integrated into a web-based platform for use in daily clinical routine.

## Results

We identified 255,837 men with newly diagnosed localized PCa who met the inclusion and exclusion criteria (Supplementary Fig. 1). Among these, 26,389 (10.3%) underwent AS/WW, 86,714 (33.9%) underwent RP, 76,919 (30.8%) underwent RT, and 63,815 (24.9%) underwent other/unknown treatments, including androgen deprivation therapy (ADT), combined ADT and RT, etc. In the low-risk cohort model (patients with clinical T stage T1c and T2a, Gleason grade group 1, and PSA ≤ 10 ng/mL, age < 80), 79,633 patients were included. Among them, 17,553 (22%) underwent AS/WW and 62,080 (78%) underwent other treatments such as androgen deprivation therapy or focal therapy.

### Entire cohort

**Figure 1:**
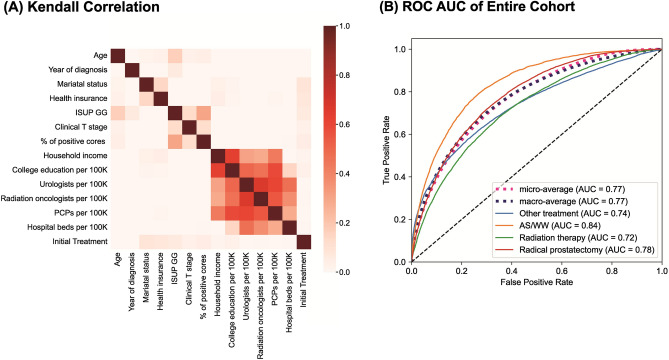
(**A**) Correlation between categorical features by Kendall method in entire localized prostate cancer cohort. (**B**) A decision prediction model using the entire cohort’s receiver operatic characteristics curve and multiclass area under the curve (AUC).

Regional factors, such as the SEER registry, state/county, average level of education, average number of healthcare providers (urologists, radiation oncologists, primary care practitioners), and health facilities showed correlative associations (Fig. 1A). The decision-predicting machine-learning model achieved fair discrimination in the test set, with an overall multiclass AUC of 0.77 (Fig. 1B). The easiest treatment to distinguish among them was AS/WW (AUC of 0.84), whereas the most difficult to distinguish was RT (AUC of 0.72). RP treatment showed intermediate discrimination performance (AUC of 0.78).

### Identification of the important features per treatment

**Figure 2:**
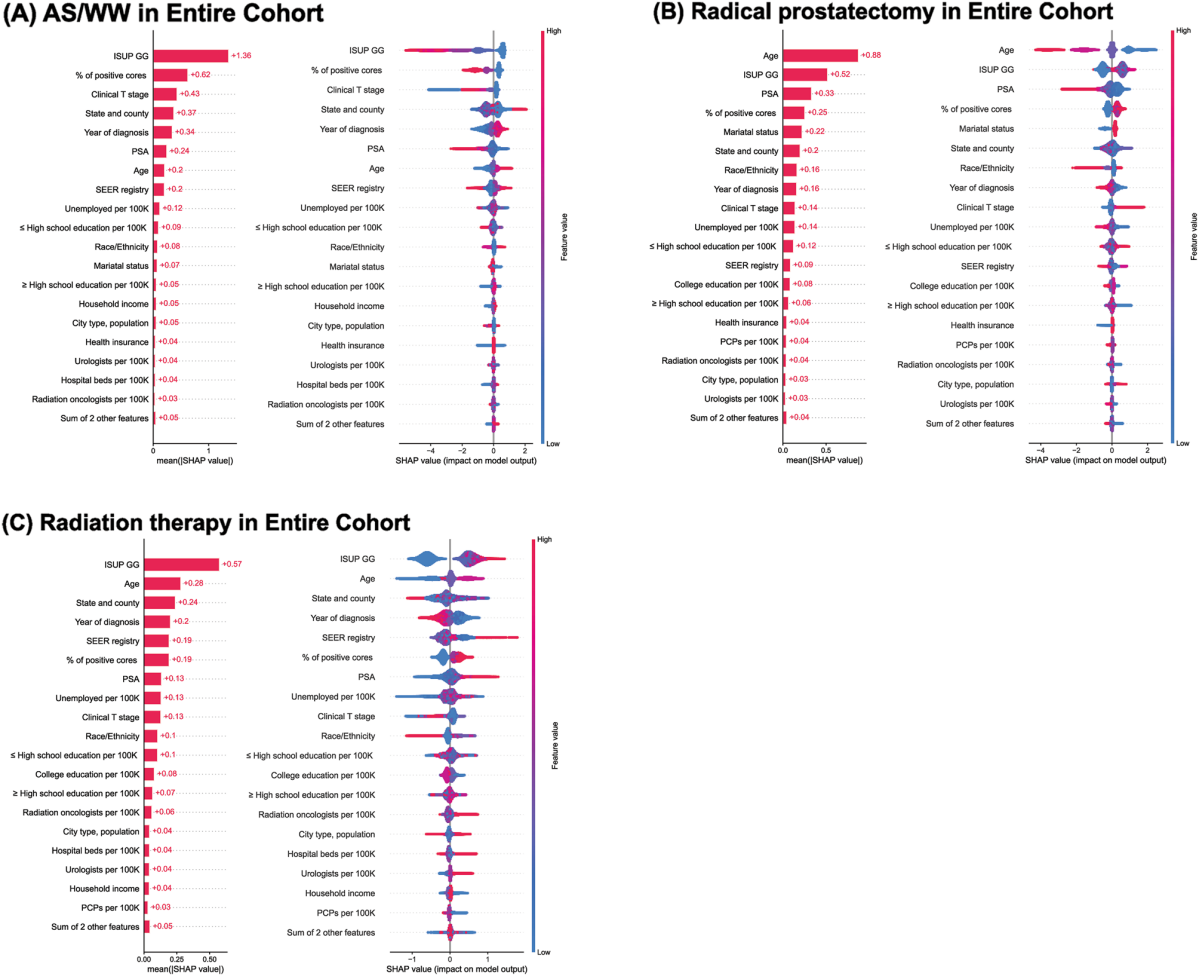
Global feature importance plot and Beeswarm plot using SHAP value for each treatment decision (**A**) AS/WW (**B**) RP (**C**) RT in the entire localized prostate cancer cohort.

The contribution and effect of each feature (as measured by SHAP scores) on the chosen outcome are presented in Fig. 2. The decision to perform AS/WW was mostly affected by the ISUP grade group (GG), followed by the positive core percentage (PPC), clinical T stage, state/county, and year of diagnosis. Those for RP were age, ISUP GG, PSA, and PPC. For RT, ISUP GG, age, state/county, and year of diagnosis were the important features.

### Interaction of features in a dependence plot

Within the AS/WW group, for the T1c stage, low PPC patients chose AS/WW, whereas T2 stage patients tended to choose AS/WW when PPC was high. Similarly, for the same ISUP GG, groups with a differing PSA level (low vs. high) showed different decision-making (Supplementary Fig. 2). For the RP group, the recently diagnosed patients chose RP when ISUP GG was high, whereas previously diagnosed patients showed the opposite trend (the ISUP GG low group chose RP). In addition, there was a race/ethnic difference in RP treatment decisions, where white men tended to choose RP when PPC was high, while those with other races avoided RP (Supplementary Fig. 3). For the RT group, ISUP GG1 preferred not to choose RT, while ISUP GG2-5 preferred RT. PSA was a distinct oncological feature that affected different decisions regarding RT treatment. The older group avoided undergoing RT when PSA was high and chose AS/WW instead, while the younger group preferred undergoing RT when PSA was high. Furthermore, T1 and T2 stage patients showed the opposite trend of RT decision for similar accompanying oncological characteristics. Typically, high PPC had a positive impact on RT decision-making in T2 stage, but a negative impact in T1 stage patients (Supplementary Fig. 4).

### Low-risk localized prostate cancer cohort

**Figure 3:**
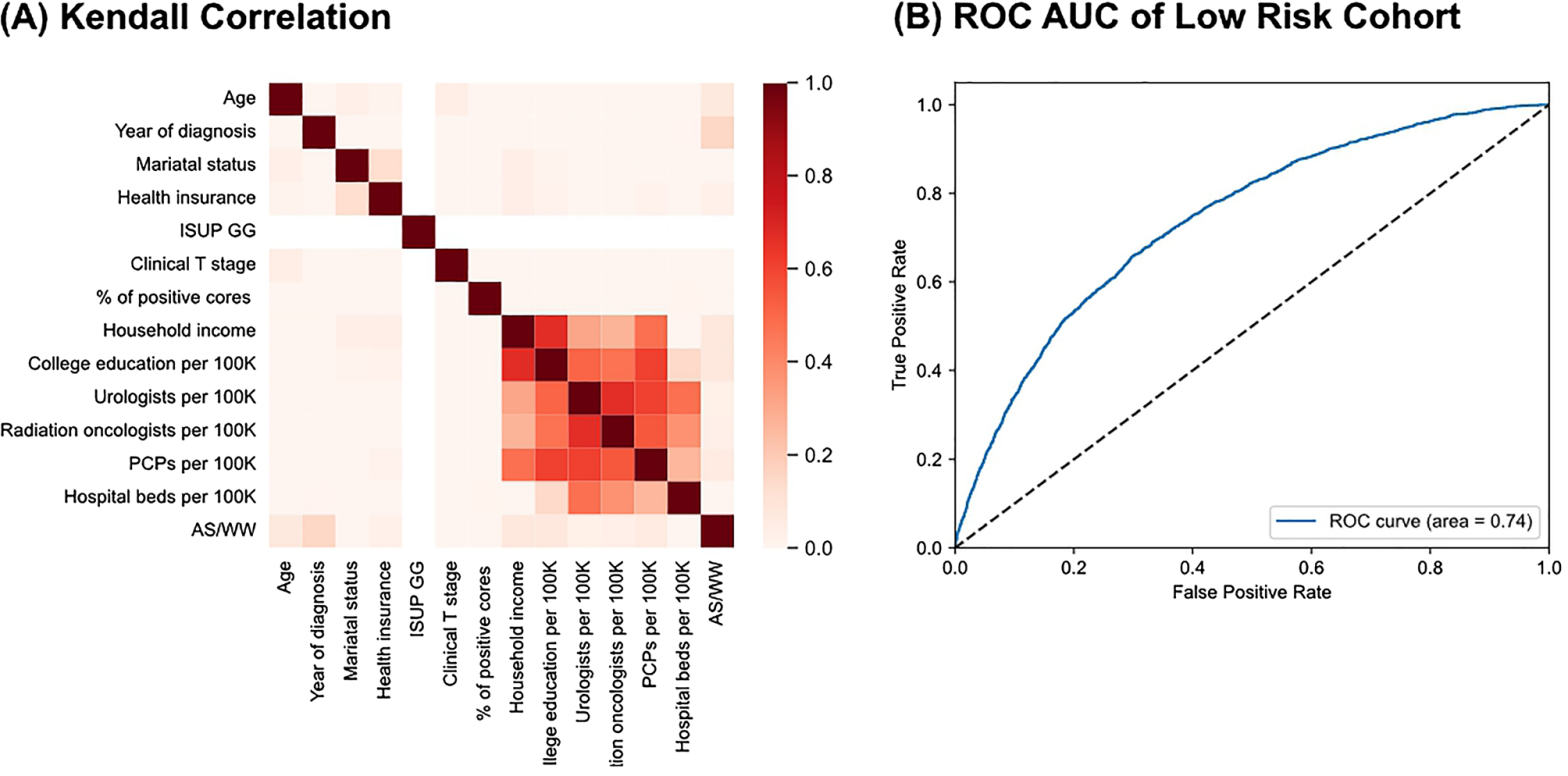
(**A**) Correlation between categorical features by Kendall method in low-risk cohort. (**B**) Receiver operational characteristics curve and multiclass area under the curve (AUC) measure in a decision prediction model with a low-risk cohort.

**Figure 4:**
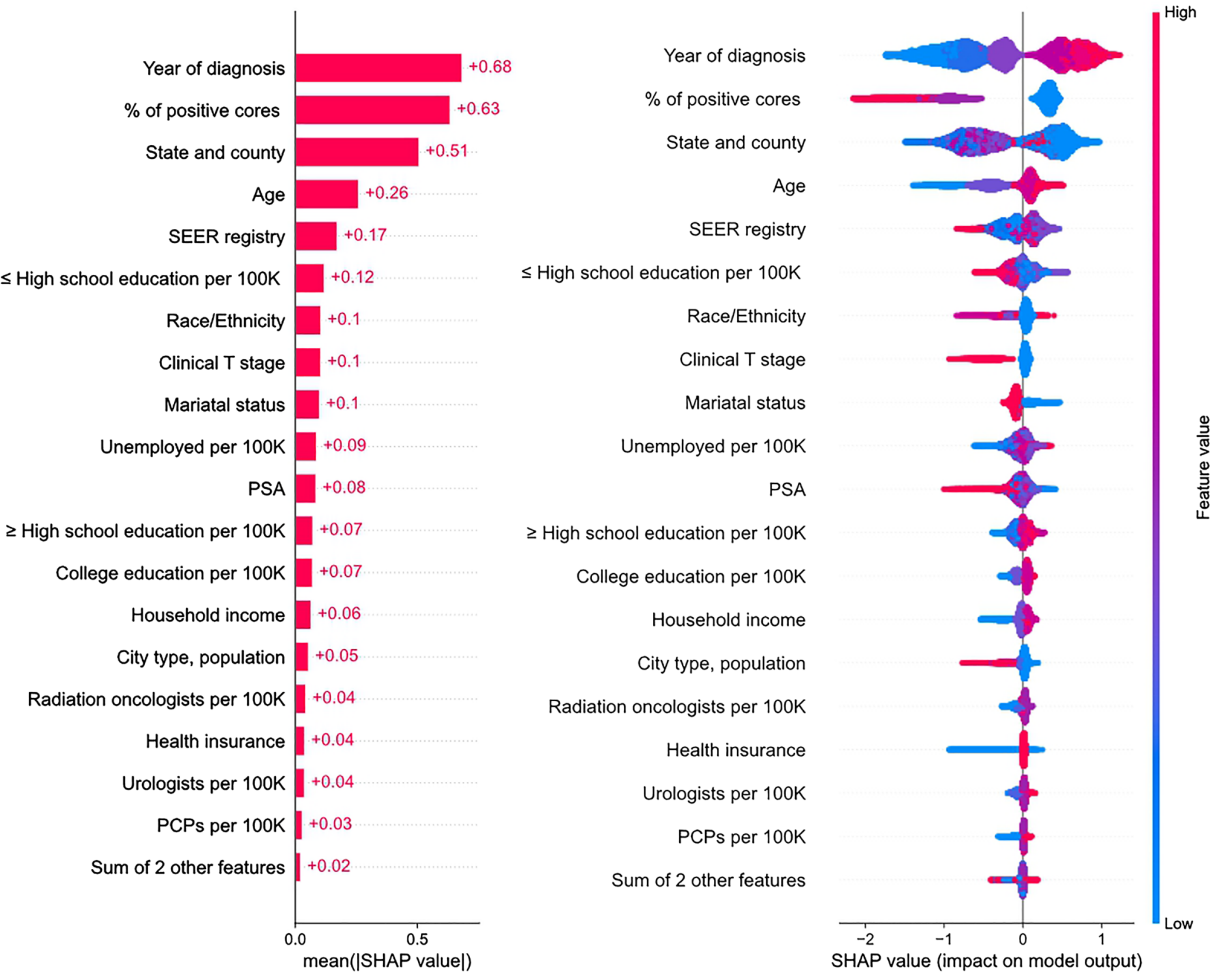
Global feature importance plot and Beeswarm plot for AS/WW decision in low-risk cohort using SHAP value.

The same correlation was observed in the low-risk cohort as in the entire cohort, showing a positive correlation between regional factors (Fig. 3A). The decision-predicting machine learning model achieved fair discrimination, with an overall binary class AUC of 0.74 (Fig. 3B). The most important factor in the AS/WW decision was the year of diagnosis, followed by the PPC, state/county, age, SEER registry, and education (Fig. 4).

### Interaction of features in the low-risk cohort model's dependence plot

There were different characteristics between races and ethnicities. White patients tended to choose AS/WW when age was high or PPC was low, while patients of other race or ethnicity (black, Hispanic, Asian, and others) showed the opposite trend. For T1c stage, recently diagnosed patients or those with higher PSA levels chose AS/WW, whereas T2a stage patients preferred active treatments when recently diagnosed or with a lower PSA level (Supplementary Fig. 5).

### Development of an online applicable prediction model using the SEER/WW platform

The model is open for patient access at http://210.117.211.210:8501/ (Supplementary Fig. 6). Model information and details are listed in Supplementary Table 2.

## Discussion

The factors that influence the complex decision-making process in the treatment of localized PCa remain debatable^10^, suggesting that we still lack an understanding of these complex processes. Thus, clinicians collaboratively help patients make proper decisions through a shared decision-making process, such as informing evidence about the safety and value of each treatment method^8^ or informing the types of treatments offered to similar men with similar cancer characteristics to have more consistency in their decisions. In this regard, the Michigan Urological Surgery Improvement Collaborative (MUSIC) recently developed a new machine learning model to help men view predicted treatment decisions of similar patients using a prospective registry of 7543 men diagnosed with prostate cancer^11^. However, this random forest model was unable to provide a distinct understanding of how a single decision was reached and it did not consider geographic and socioeconomic variations in the treatment decision^12^.

To compensate for these shortcomings, we adopted an explainable machine learning model using the recently released SEER-WW dataset (2010–2016), which has a newly created variable clearly defined as “AS/WW”. Precise model training was enabled by a more accurate treatment group classification compared with the existing SEER databases. We trained machine learning models using a broad range of real-world features as input, including clinicopathologic characteristics as well as demographics, socio-economic information, and nationwide county level geographic data, including regional healthcare resources, which the MUSIC group (Michigan state-confined cohort) could not address. Usually, there is a trade-off between interpretability and accuracy for each machine learning model^13^; however, our model showed interpretability while having comparable model performance with the MUSIC group (AUC 0.77 vs. 0.81).

The overall characteristics of the machine learning model developed in this study included geographic features (state/county and SEER registry) that mostly affected RT treatment decisions, followed by AS/WW. This result agrees with a previous study by Wang et al. who showed that the availability of RT is the most important contributor to regional variation^4^. Meanwhile, a previous paper by Washington et al. demonstrating that geographic location is associated with AS/WW practice variation^6^, better explains the regional factors influencing AS/WW decisions. Contrary to our expectations, socio-economic features (marital status, educational status, household income, insurance, etc.) generally did not influence treatment decision-making, except that in RP patients, married men showed a tendency to undergo surgery compared to unmarried/single men. This likelihood is commonly observed in several other cancers (lung cancer, breast cancer, etc.)^6,12,14^, and Schymura et al. demonstrated that unmarried men are more likely to choose conservative treatment or RT rather than RP^15^, which is consistent with our results.

Cancer characteristics (ISUP GG, PPC, clinical T stage, PSA, etc.) contributed significantly to AS/WW or RP treatment decisions, in contrast to RT treatment decisions. Age was the most important feature in active treatment decision-making (first in RP and second in RT), likely because of its correlation with accompanying comorbidities and life expectancy^16^. Consistent with previous data demonstrating the impact of ethnicity on PCa treatment decisions using logistic regression models^12^, we observed a racial/ethnicity difference in the initial treatment. However, its impact on the overall model performance was relatively low, ranging from the seventh to tenth ranked feature. Finally, the year of diagnosis was the most important feature in deciding AS/WW in the low-risk model, demonstrating the current time trend of an increasing proportion of AS/WW patients^17^. In other words, time trend reflects evolving evidence for AS/WW.

Several notable and solid relationship visualizations add further depth to this study. In the AS/WW treatment outcome model, the older (≥ 70 years) and younger age groups demonstrated different decision-making. The older group with higher oncological risk (high PSA or PPC or ISUP GG) chose AS/WW, while the younger group did not choose AS/WW in the same condition and underwent definitive treatment (Supplementary Fig. 2). This result is consistent with the overall feature contribution result that age was the most important feature in deciding active treatments (RP and RT). Furthermore, racial/ethnic differences were observed in the older age group. While older white men tended to pursue AS/WW, other races largely avoided it, which was more clearly identified in the low-risk cohort model (Supplementary Fig. 5). This may indicate that white men tend to emphasize treatment burden, while other races emphasize treatment efficacy/cure^18^, especially in the low-risk group. This cohort of patients was unlikely to progress and may not require radical treatment^19^. We also observed that patients who are presumed to have a large tumor burden (PPC ≥ 50%) avoided both RP and RT monotherapy treatment, and chose androgen-deprivation therapy or combinatory treatment when accompanied by other poor prognostic oncological features, such as high ISUP GG or high PSA.

With the aid of year of diagnosis variable annotation, we visually inspected the time trend of the associated variables. Although there was a trend of increasing AS/WW decision making, recently diagnosed patients chose not to undergo AS when high ISUP or PPC were present, which was in contrast to the previous period (Supplementary Fig. 2), possibly because of the recent publication of AS criteria^20^. Regarding the active treatment groups, although both have been decreasing recently, patients with higher oncological risk (high ISUP GG, high PPC, or high PSA) more actively tended to decide on active treatment, which is consistent with previous data showing an increasing trend of RP in the intermediate-to-high-risk group^17^.

This study has several limitations. First, SEER database only covers 30% of US cancer patients, raising concerns about its generalizability. Furthermore, the SEER and Area Health Resource File (AHRF) dataset do not include the same patient groups, introducing potential issues during data integration. However, SEER database is still the most well-established population-based epidemiologic cohort, and the AHRF data were linked to the county-level data where the patients reside within the SEER/WW dataset, utilizing a matching process based on the combined Federal Information Processing System (FIPS) codes for states and counties. To enhance the regional matching accuracy, year matching was also implemented. Second, the outcome of this predictive model represents the complicated treatment decision result; thus, it may be different from the decision prediction. Third, although data preprocessing was done to reduce the issue of feature imbalance, the potential clinical bias still remained. For example, as this cohort was developed in the active PSA screening era, the cohort distribution was heavily skewed towards earlier stage disease. Fourth, external validation was lacking. Fifth, there may be unidentified confounding factors in the analysis of the interaction dependence plot that hinder the ability to reach firm conclusions. Sixth, several important factors, such as life expectancy, preexisting urinary symptoms, genomic data (somatic and/or germline), family history, and types of health insurance could not be addressed due to the unavailability in SEER/WW dataset. Seventh, for patients classified into the observation group, WW or AS could not be discriminated, although they are completely different treatment approaches. To address this issue, we utilized explainable interaction plots to approximate the distinction between the two groups. And eighth, patient population were enrolled between 2010 and 2016, potentially preventing the reflection of the most recent trends. Also, there may be a drawback that the latest AS inclusion criteria were not applied to these patients. However, it is worth noting that in first decade of 2000, significant consensus was reached on the establishment of AS criteria and suggested as guideline, which is almost equivalent to the current standards^21^.

Despite these limitations, this study demonstrates important clinical implications. First, with the help of the explainable SHAP method and further descriptive interaction plots, we extracted a plausible description of each treatment decision in an orthogonal manner, leading to a comprehensive understanding and deeper insight. Second, our machine-learning-based model was trained on a large, contemporary, ethnically heterogeneous population using real-world data from a high-quality database^22^. Third, the potential power of this approach (offering decisions made by similar men) has been proven in a previous patient-led online community study by helping patients understand the decision-making process^23^. Fourth, compared to a similar study performed by the MUSIC group, the predictive model in this study was fitted with additional non-oncological features, such as socioeconomic and geographical regional factors. Geographic features affected RT treatment decisions to the greatest degree.

## Conclusions

Using a large population-based real-world database, we could have a deeper insight into the complex decision-making process and visualize nonlinear feature interactions in localized PCa.

## Subjects and methods

Supplementary Fig. 7 provides a summary of the analysis. The experimental dataset utilized in this study consists of the clinical SEER/WW dataset and the regional County AHRF dataset. To improve the classification performance, preprocessing techniques were taken to address class imbalance, as well as to mitigate the presence of outliers or noisy data. The preprocessed dataset was then divided into training, validation, and test sets. The classification model was trained iteratively using the training set, aiming to identify the optimal machine learning algorithm and corresponding hyperparameters. The model's optimality was evaluated using the validation set. Finally, model explanation techniques were applied to gain insights into feature importance and interactions. The rest of this section includes more detailed information.

### Study cohort

For the experimental analysis, we used the following two datasets: (1) the SEER/WW dataset (2010–2016) which had undergone multiple imputations for missing data handling^24^, and (2) the AHRF, which includes data on variable health care-related features, such as healthcare providers by specialty, health facilities, population demographic characteristics, income, and hospital utilization^6^. We merged these two datasets in one final experimental dataset (n = 255,837). Supplementary Table 1 shows the labels encoding the correspondence between categorical variables, including nominal variables. Initial treatment variable was divided into four groups: AS/WW, RT, RP, and other/unknown treatments. We included men with concurrent RP and RT treatment into the other treatment group as a small number of RP + RT class could skew the whole class distributions. The low-risk cohort was refined to include patients with clinical T stage T1c and T2a, Gleason grade group 1, and PSA ≤ 10 ng/mL, Men aged > 80 years were excluded from the study, consistent with an appropriate patient population for AS^6^. The initial treatment features in the low-risk group were relabeled into two classes: AS/WW (AS + WW) and other treatments (Supplementary Fig. 1).

The Seoul National University Hospital institutional review board deemed this study exempt from review and informed consent because patient information in these databases was completely de-identified and publicly available.

### Data preprocessing

Oversampling and undersampling techniques were adopted to circumvent the class imbalance problem. MSMOTE (Modified SMOTE)^25^ as an oversampling method, was primarily applied to consider the distribution of minority class instances and remove noisy instances. For the undersampling method, we used the edited nearest neighbor (ENN)^26^, which is based on a nearest-neighbor algorithm to remove samples whose class differs from the majority class of their neighborhood in a broad sense.

### Model development

We split the dataset into training (70%), validation (15%), and test (15%) sets. The validation set was allocated to search for appropriate hyperparameters of the model. We trained three different gradient boosting decision tree algorithm-based ensemble method models, eXtreme Gradient Boosting (XGBoost)^27^, LightGBM^28^, and CatBoost^29^, and found that the XGBoost model had the highest explanatory power in our experimental setting.

### Model validation

After fitting the XGBoost model using the training set, the discrimination of the model was evaluated in the test set using a multiclass area under the curve (AUC) measure. Model calibration was evaluated using a calibration plot that compared the predicted classes for each outcome with the observed classes.

### Model explanation

SHapley additive exPlanations (SHAP)^30^ serves two functions. (1) To demonstrate the contribution of each feature to the model’s overall prediction results, visualizing with the global feature importance and Beeswarm plots. (2) To unravel the nonlinear complicated relationship between two principal features using a two-way dependence scatter plot and make it possible to gain deeper insight into the complex decision process.

### Development of the web platform

We developed the Proca (Prostate Cancer treatment Advisor; Slogan: Friendly advise from patients like you) web platform based on Streamlit, an open-source Python library for building data apps. The web platform shows the initial treatment prediction for a patient using the given inputs. The inputs consisted of demographic and clinical information that appeared during the experiment. Furthermore, the web platform presents several important factors that influence the prediction. This tool is predictive of patient choice in a given scenario, so would suggest what other people have chosen. This is not about what each patient should do for their localized PCa.

### Software

R version 3.6 to perform multiple imputation and statistical analysis for the completion of the experimental dataset. Python 3.8.10, and XGBoost 1.4.2 were used for the model algorithm. Train/test split, shuffle, and other data preprocessing procedures were conducted using Scikit-learn 0.24.2. Smote-variants 0.4.0 were used to address the class imbalance problem. Finally, the shap 0.39.0 library was employed for model interpretation.

## Supplementary Information

Supplementary Information.

## Data Availability

The datasets generated during and/or analysed during the current study are available from the corresponding author on reasonable request.
