# Supplementary material for: Explainable ML models for a deeper insight on treatment decision for localized prostate cancer
Source: Sci Rep. 2023 Jul 17;13:11532. doi: 10.1038/s41598-023-38162-1 (PMC10352331; doi:10.1038/s41598-023-38162-1)
Supplement: Supplementary file 1 — Supplementary Information. [file 41598_2023_38162_MOESM1_ESM.docx]

**Supplementary Figure 1. Flow chart of patients meeting the inclusion and exclusion criteria.**

SEER-WW denotes the SEER prostate database with watchful waiting AHRF denotes the County Area Health Resource File.


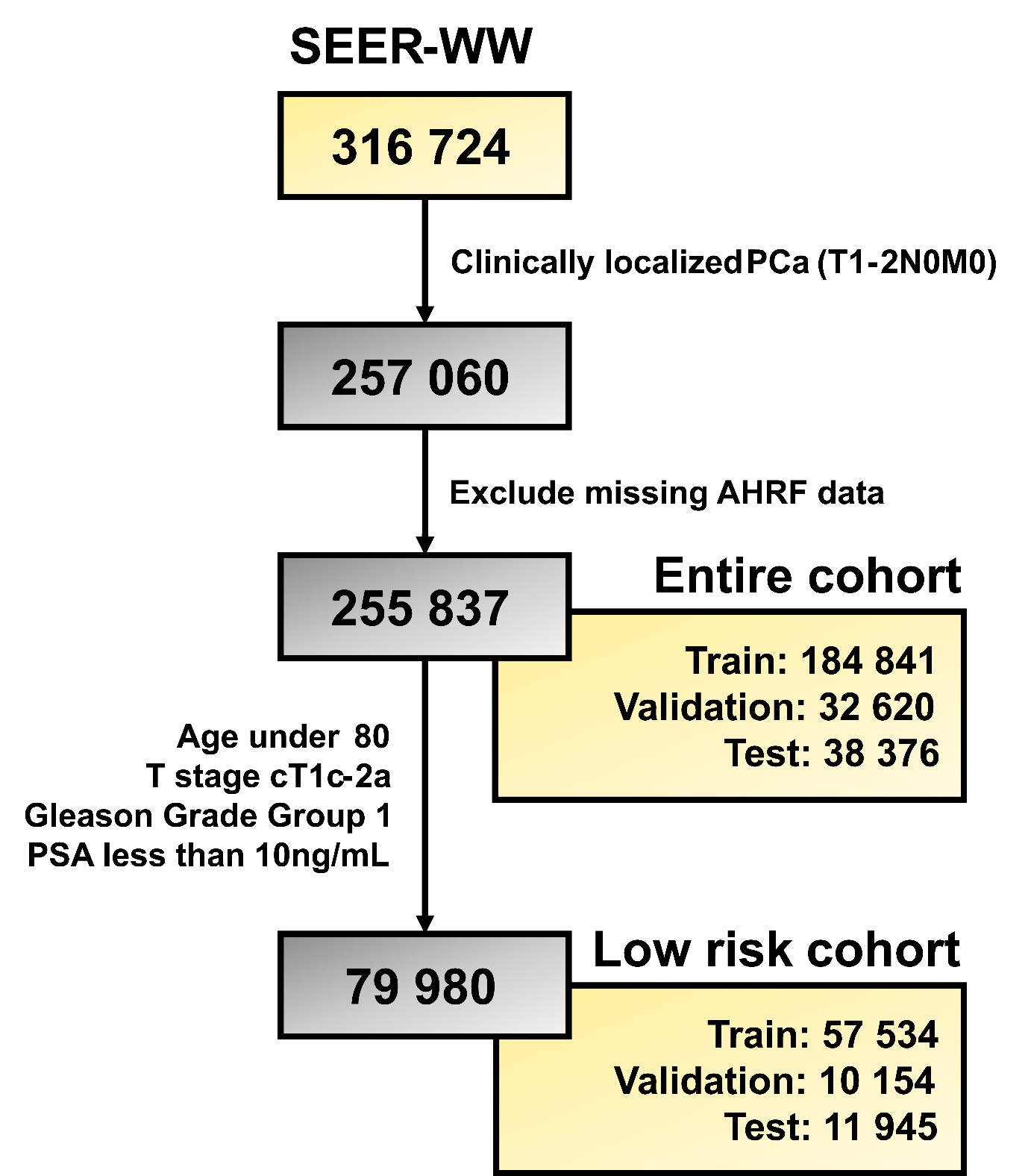


**Supplementary Figure 2. Interaction plot in the AS/WW decision in the entire cohort.** The color bar indicates the SHAP value, while the gray histogram demonstrates the population distribution for each factor.


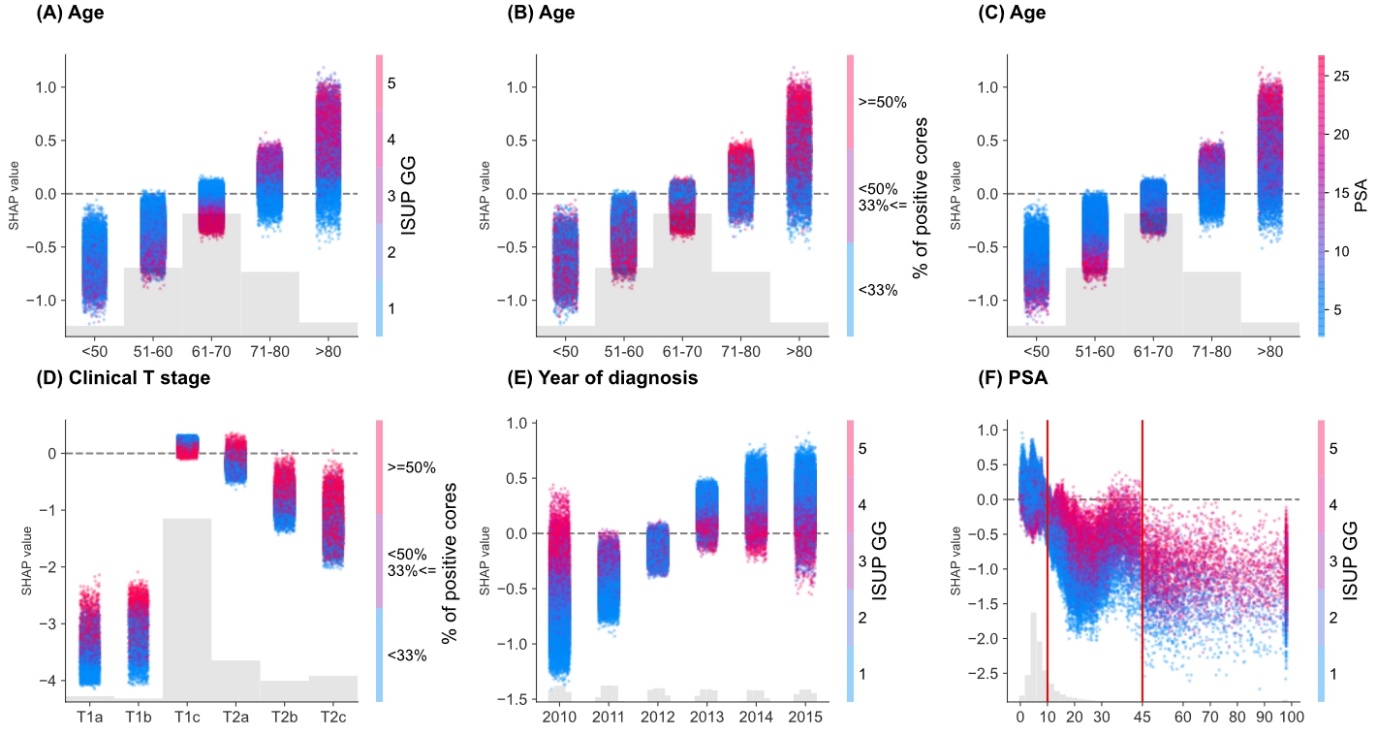


**Supplementary Figure 3. Interaction plot in RP decision across the entire cohort.**

The color bar indicates the SHAP value, and the gray histogram shows the population distribution for each factor.


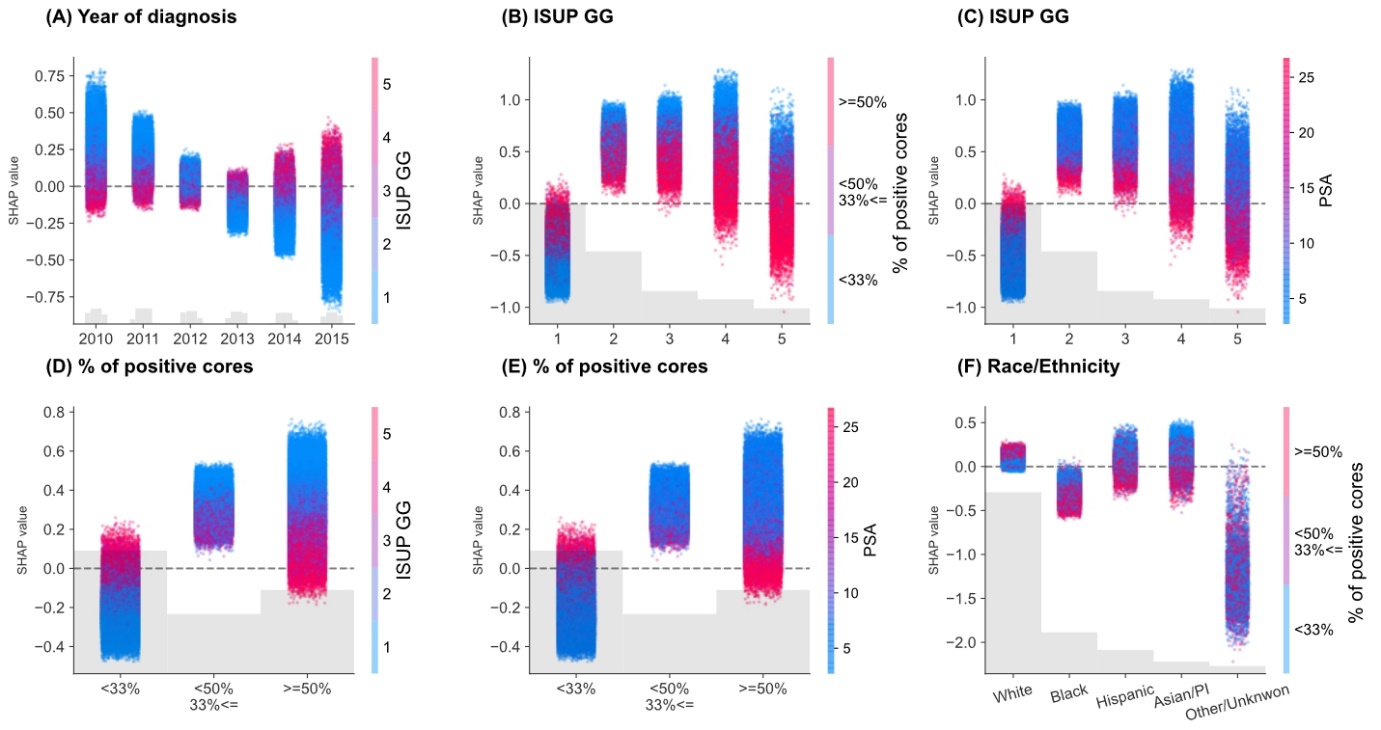


**Supplementary Figure 4. Interaction plot in RT decision across the entire cohort.**

The color bar indicates the SHAP value, and the gray histogram shows the population distribution for each factor.


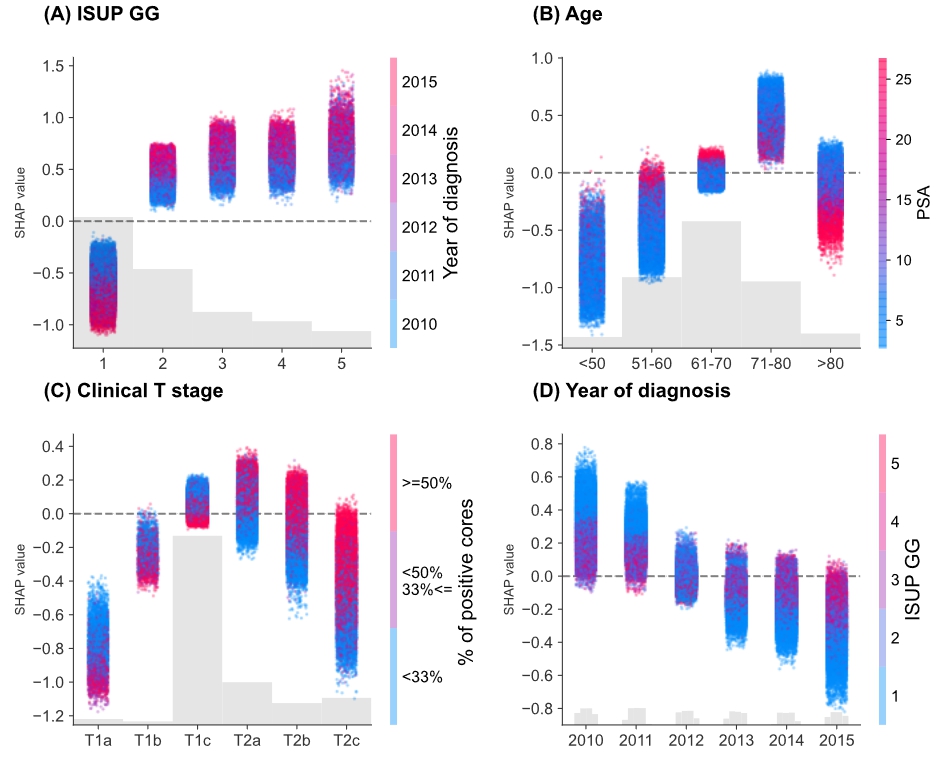


**Supplementary Figure 5. Interaction plot in AS/WW decision in low-risk cohort.**

The color bar indicates the SHAP value, and the gray histogram shows the population distribution for each factor.


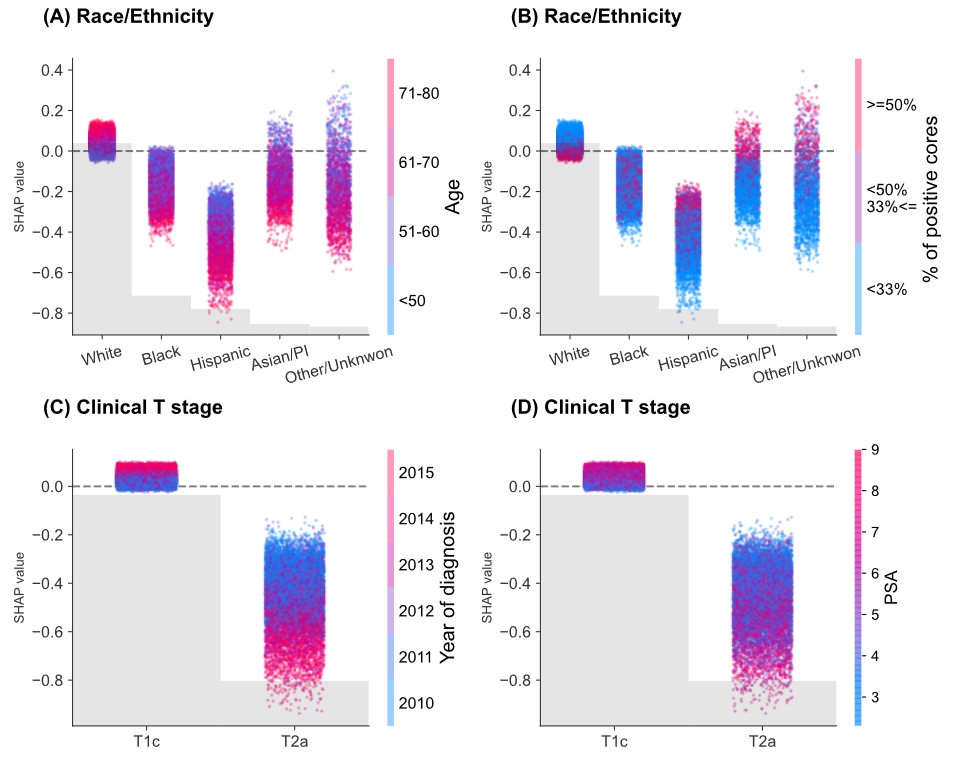


**Supplementary Figure 6. Screenshot of the Proca web platform.**

**
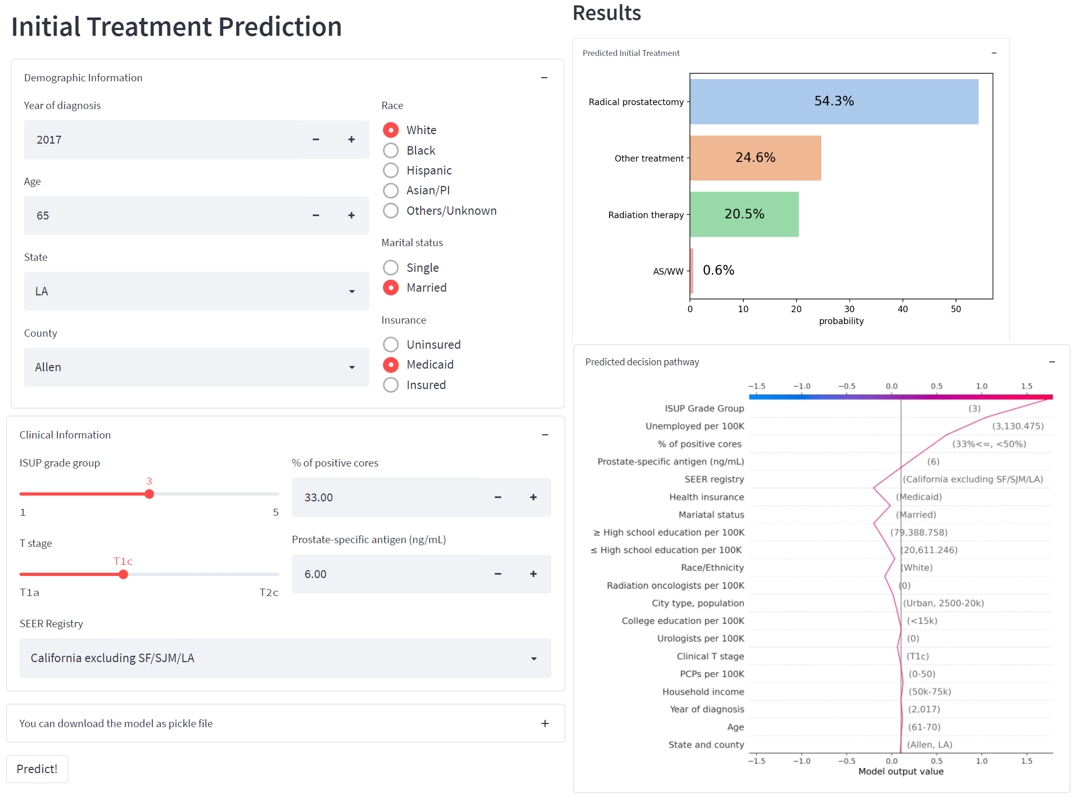
**

**Supplementary Figure 7. Graphical summary of analysis method.**


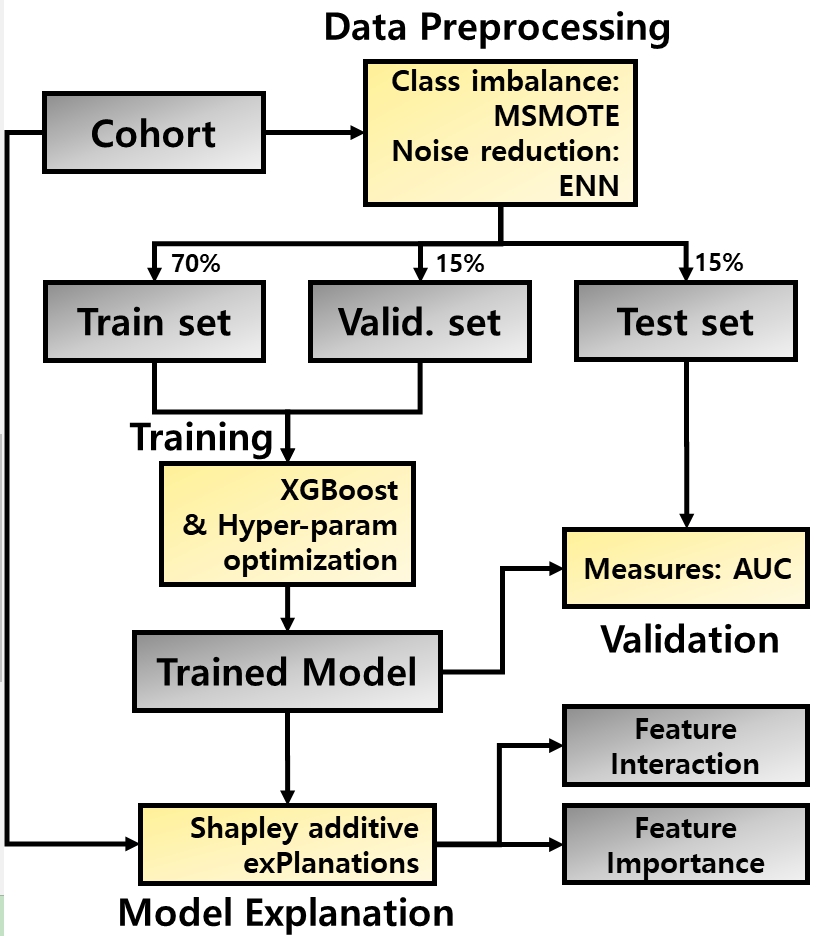


**Supplementary Table 1. Coding table for categorical variables**

| **Description** | **Coding** |
| --- | --- |
| Age at diagnosis | 1. <50, 2. 51-60, 3. 61-70, 4. 71-80, 5. >80 |
| Race/Ethnicity | 1. White, 2. Black, 3. Hispanic, 4. Asian/PI, 5. Others/Unknown |
| Gleason grade group | 1, 2, 3, 4, 5 |
| T stage category | 1. T1a, 2. T1b, 3. T1c, 4. T2a, 5. T2b, 6. T2c |
| PPC | 1. <33%, 2. >=33%, <50%, 3. >=50% |
| Marital status | 1. Married, 0. Single |
| Insurance status | 2. Insured, 1. Medicaid, 0. Uninsured |
| Year of diagnosis | 2010, 2011, 2012, 2014, 2015 |
| Median household income in the county in $ | 1. <50k, 2. 50k-75k, 3. 75k-100k, 4.>100k |
| College education per 100k | 1. <15k, 2. 15-30k, 3. 30-45k, 4. >45k |
| City type, population | 1. Metropolitan, >1M, 2. Metropolitan, 250k-1M, 3. Metropolitan, <250k, 4. Urban, >20k, 5. Urban, 2500-20k, 6. Rural/Urban, <2,500 |
| Number of urologists per 100k | 1. 0, 2. 0-2, 3. 2-4, 4. 4-6, 5. >6 |
| Number of radiation oncologists per 100k | 1. 0, 2. 0-1, 3. 1-2, 4. 2-3, 5. >3 |
| Number of primary care practitioner per 100k | 1. 0, 2. 0-50, 3. 50-100, 4. >100 |
| Number of hospital beds per 100k | 1. 0, 2. 0-200, 3. 200-400, 4. 400-600, 5. >600 |
| SEER registry (region) | 1. California excluding SF/SJM/LA, 2. New Jersey, 3. Greater Georgia, 4. Los Angeles, 5. Louisiana, 6. San Francisco-Oakland SMSA, 7. Detroit, 8. Seattle, 9. Kentucky, 10. Connecticut, 11. Atlanta, 12. San Jose-Monterey, 13. Iowa, 14. Utah, 15. New Mexico, 16. Hawaii, 17. Rural Georgia |
| State and county | Federal Information Processing System (FIPS) code |
| Initial treatment | 1. AS+WW, 3. Radical prostatectomy, 2. Radiation therapy, 0. Other treatments (including RP+RT) |

**Supplementary Table 2. Model details**

| **Model information** | **Web location** | **Comments** |
| --- | --- | --- |
| **Model location for patient use** | http://210.117.211.210:8501/ | The model is also downloadable. |
| **Model as Pickle** |  | For more details on Pickle, see here: <https://docs.python.org/3/library/pickle.html> |
| **Model details** | *XGBoost model*  Number of trees: 3636  Tree depth: 5  Minimum leaves/tree: 8  Maximum leaves/tree: 32  Mean number of leaves/tree: 27 | The model was trained using XGBoost version 1.4.2. Details on XGBoost are available here:  [XGBoost Documentation — xgboost 1.5.2 documentation](https://xgboost.readthedocs.io/en/stable/) |
